# Mark Twain’s Tribute to Homœopathy

**Published:** 1890-05

**Authors:** 


### MARK TWAIN'S TRIBUTE TO HOMŒOPATHY.

In an article, contributed to *Harper's Monthly* for February, to which we have before called the attention of our readers in the March number, p. 141, Mark Twain writes, half humorously and half in earnest, upon "A Majestic Literary Fossil," and pays a deserved compliment to Homœopathy for the purifying work it has done in medicine. And this work has been done by pure Hahnemannian Homœopathy; not in the least by its weak eclectic imitator. That the compliment is deserved on the part of Homœopathy, any one may convince himself by studying the works upon both practice and materia medica, published and used by the old school during the past fifty years. In the earlier works he will read of all these ancient methods, which Mark Twain justly ridicules; in the works, recently issued, he will see crude imitations of homœopathic practice. The change has been not only in the amounts, the complexity, and the character of the ingredients used in their prescriptions, but also in the manner in which drugs are prescribed. As Mark

Twain says, this change has been an utter reversal, in a couple of generations, of an attitude which had been maintained without challenge or interruption from earliest antiquity. He writes:

"So recent is this change from a three or four thousand year twilight to the flash and glare of open day that I have walked in both, and yet am not old. Nothing is to-day as it was when I was an urchin; but when I was an urchin, nothing was much different from what it had always been in this world. Take a single detail, for example—medicine. Galen could have come into my sick-room at any time during my first seven years —I mean any day when it wasn't fishing weather, and there wasn't any choice but school or sickness—and he could have sat down there and stood my doctor's watch without asking a question. He would have smelt around among the wilderness of cups and bottles and phials on the table and the shelves, and missed not a stench that used to gladden him two thousand years before, nor discovered one that was of a later date. He would have examined me, and run across only one disappointment—I was already salivated; I would have him there; for I was always salivated, Calomel was so cheap. He would get out his lancet then; but I would have him again; our family doctor didn't allow blood to accumulate in the system. However, he could take dipper and ladle, and freight me up with old familiar doses that had come down from Adam to his time and mine; and he could go out with a wheelbarrow and gather weeds and offal, and build some more, while those others were getting in their work. And if our reverend doctor came and found him there, he would be dumb with awe, and would get down and worship him. Whereas if Galen should appear among us today, he could not stand anybody's watch; he would inspire no awe; he would be told he was a back-number, and it would surprise him to see that that fact counted against him, instead of in his favor. He wouldn't know our medicines; he wouldn't know our practice; and the first time he tried to introduce his own, we would hang him."

[After giving many examples of ancient practice, with its crude ideas, its horrible mixtures, etc., the writer concludes by declaring :]

"When you reflect that your own father had to take such medicines as the above, and that you would be taking them to-day yourself but for the introduction of Homœopathy, which forced the old-school doctor to stir around and learn something of a rational nature about his business, you may honestly feel grateful that Homœopathy survived the attempts of the allopathists to destroy it, even though you may never employ any physician but an allopathist while you live."

If Homœopathy has done so much for mankind, is it not worth preserving in its purity and strength? E. J. L.

